# Changes in Tinnitus Characteristics and Residual Inhibition following Cochlear Implantation: A Prospective Analysis

**DOI:** 10.3390/brainsci13101484

**Published:** 2023-10-20

**Authors:** Ann Nancy Deklerck, Freya Swinnen, Hannah Keppler, Ingeborg Johanna Maria Dhooge

**Affiliations:** 1 Department of Otorhinolaryngology, Faculty of Medicine and Health Sciences, Ghent University, 9000 Ghent, Belgium; ann.deklerck@ugent.be; 2 Department of Rehabilitation Sciences, Faculty of Medicine and Health Sciences, Ghent University, 9000 Ghent, Belgium; freya.swinnen@ugent.be (F.S.); hannah.keppler@ugent.be (H.K.); 3 Department of Otorhinolaryngology, Ghent University Hospital, 9000 Ghent, Belgium

**Keywords:** cochlear implantation, tinnitus, neuromodulation

## Abstract

This study aims to explore the effect of cochlear implantation on tinnitus perception. A prospective study was conducted on 72 adult hearing-impaired patients to evaluate tinnitus perception before and after cochlear implantation, using standardized tinnitus questionnaires (the tinnitus sample case-history questionnaire, tinnitus functional index (TFI), and tinnitus handicap inventory (THI)). A large variety of demographic and hearing- and implant-related data was collected from patient hospital records to explore possible associations with the implantation effect. The prevalence of tinnitus complaints before implantation was 58.3%. The temporary induction or aggravation of tinnitus immediately after surgery was noted in 20% and 46.7% of patients, respectively. When evaluated 3 months after implantation, 60% of tinnitus patients experienced a clinically significant reduction in their complaints; most of the improvements were experienced immediately after activation of the implant. Only the scores for TFI and THI at baseline were found to be significantly correlated with a reduction in TFI scores after implantation. In 80% of tinnitus patients, the tinnitus remained suppressed for some time after taking off the device. The large subset of patients with residual inhibition supports the involvement of central pathophysiological processes in implantation effects on tinnitus, which are explored in this paper.

## 1. Introduction

The prevalence of tinnitus in the general population is reported to be 10–15% [1,2,3,4]. However, tinnitus is present in approximately 80% of patients with bilateral profound sensorineural hearing loss (SNHL) [5], and tinnitus is reported in 66–100% of adult patients receiving cochlear implantation (CI) [6,7,8,9,10,11,12].

As CI is considered the standard treatment for bilateral profound hearing loss [13], reports have been published on the effect of implantation on accompanying tinnitus complaints. Starting from the very first report in 1976 [14], divergent reduction effects of CI on concomitant tinnitus perception have been reported [6,15,16,17]. The effect of implantation on reducing tinnitus perception ranges from 46 to 95% [7,10,15,16] and from 15 to 83% regarding total tinnitus suppression [9]. This beneficial effect has led to the use of CI for unilateral hearing loss (single-sided deafness, or SSD), with tinnitus as the primary indication [18,19,20,21]. CI may, thus, be seen as an effective tinnitus treatment strategy for patients with severe SNHL. However, the worsening of tinnitus post-implantation (4–26%), as well as the occurrence of new tinnitus complaints post-surgery (0–23.5%) [7,8,21,22,23], have been reported as well.

The limitations of previous research indicate the need to address these bottlenecks: many studies have adopted a retrospective or cross-sectional design using non-validated measuring tools (e.g., patient reports instead of the tinnitus functional index (TFI) [24,25,26]), and only a few studies have investigated possible effect-associated demographic, implant-, tinnitus-, or hearing-related factors [8,10,11,27,28]. Most studies focus on subjects with single-sided deafness rather than on the classic candidates for CI: those with bilateral severe to profound SNHL [18].

The objectives of the current study were, first, to investigate the possible effects of CI on tinnitus (suppression, aggravation, and induction) in a prospective way, studying a patient group implanted according to classic reimbursement criteria. Second, our goal was to collect quantitative and effect-sensitive data by using the tinnitus functional index (TFI) [29] for tinnitus evaluation pre- and post-implantation. Also, a large variety of possible effect-associated factors were included for analysis, including sociodemographic and hearing-, implant-, and tinnitus-related factors. Finally, we wanted to describe possible implant-induced changes that could underlie tinnitus modulation post-implantation.

## 2. Materials and Methods

### 2.1. General Study Design

This prospective, mono-centric study included 72 adult patients receiving a CI at Ghent University Hospital, irrespective of their tinnitus complaint prior to implant. The subjects were primarily implanted for uni- or bilateral severe-to-profound SNHL, whether congenital or acquired. Of these 72 patients, 5 exhibited SSD and 67 exhibited bilateral severe-to-profound SNHL. Their accompanying tinnitus complaints were assessed by presenting tinnitus-specific questionnaires (see Section 2.2, Questionnaires), given at 3 test time points: at baseline (during the pre-implantation assessment) and at 3 and 6 months post-implantation. Furthermore, demographic, hearing-related, and implant information were gathered from the patients’ hospital files.

Implantation was performed by the same surgeon for all patients. Different types of electrodes (i.e., straight or modiolus-hugging) from Cochlear, Advanced Bionics, Oticon, or MedEL were used.

### 2.2. Questionnaires

To assess the characteristics and severity/handicap of tinnitus complaints, each patient (irrespective of the presence or absence of a tinnitus complaint) completed a validated Dutch-language version of the tinnitus sample case-history questionnaire (TSCHQ) [30], the tinnitus handicap inventory (THI) [31], and the tinnitus functional index (TFI) [25,32] at baseline and at 3 months postoperatively. Non-applicable questions (e.g., for patients without tinnitus) were left blank. Patients with tinnitus at baseline or complaining of newly induced tinnitus after implantation were given questionnaires again at 6 months post-implantation. Those patients who did not report tinnitus complaints at baseline and at the first evaluation 3 months after implantation were not invited for an evaluation at 6 months postoperatively. During the evaluations, patients were also questioned about the exact timing of suppressed, newly induced, or aggravated tinnitus, the masking of their tinnitus by environmental sounds, and the occurrence of residual inhibition (RI) after removal of the CI (or hearing aid at baseline). RI was reported as being present as soon as a mitigating effect on tinnitus loudness was experienced. This could range from a small reduction in intensity to a complete suppression of perception. Tinnitus patients were divided into 2 groups post-implantation: ‘remitters’ and ‘nonremitters’, according to the difference in their total TFI score. If the patient exhibited a positive score difference of 13 points or more [25] at 3 months post-surgery compared with baseline, this was labeled as a clinically significant difference, and the patient was categorized as a ‘remitter’.

### 2.3. Demographic and Hearing- and Implant-Related Factors

Patient age, sex, hearing-loss etiology, onset, laterality, and duration information were collected from some items of the TSCHQ or from patient files and were used for analysis. The type of electrode and its effect on tinnitus, as well as whether a bimodal fitting was used, were also analyzed.

Unaided hearing thresholds were collected at baseline and at one month postoperatively to assess residual hearing and hearing preservation, respectively. Aided hearing thresholds for the implanted ear were noted at the 6-month follow-up, together with speech audiometry.

### 2.4. Statistical Analysis

Data processing and statistical analysis were performed with SPSS version 23.0 (SPSS IBM Inc., Chicago, IL, USA). For all statistical analyses, a significance level of 0.05 was used, unless otherwise indicated. Descriptive parameters were calculated, and tests of normality (i.e., the Shapiro–Wilk test, histograms, Q-Q plots, and box-and-whisker plots) were applied to ensure the correct use of parametric versus nonparametric tests. For inferential statistics, Fisher’s exact and *χ*^2^ tests were used to compare categorical variables, and Student’s *t*-test and the Mann–Whitney U test were used for the parametric and nonparametric testing of 2 independent samples of continuous variables, respectively. The correlation between possible associated variables was quantified using the Spearman correlation coefficient. To assess the variability of the THI and TFI scores over time, the nonparametric Friedman test was applied. Post hoc pairwise comparisons were carried out using the Wilcoxon signed-rank test with the Bonferroni correction applied, resulting in a significance level set at *p* < 0.017.

## 3. Results

### 3.1. Study Population

**Figure 1:**
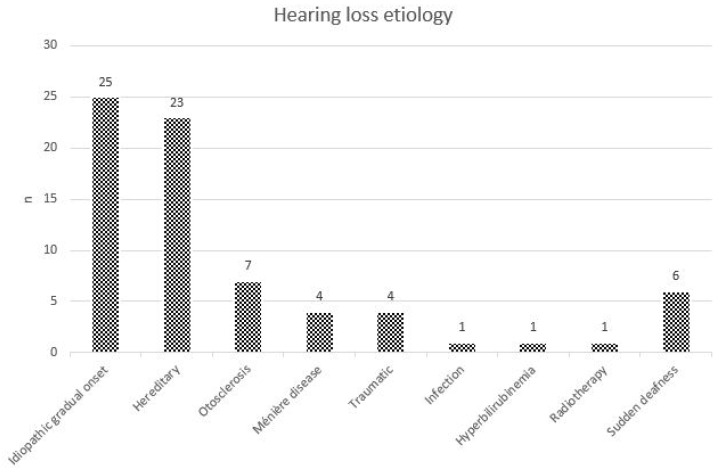
Distribution of hearing-loss etiologies in the study population.

In total, 72 subjects with a mean age of 55.7 years (±17.3, range 20 to 85 years) were included in the study. Forty subjects were female (55.6%). The average duration of hearing loss was 26.3 years (±15.2, range 3 to 60 years), and most of the study subjects exhibited a post-lingual hearing loss onset (72.2%, *n* = 52). The distribution of hearing-loss etiologies is displayed in Figure 1.

### 3.2. Characteristics of the Tinnitus Population

**Figure 2:**
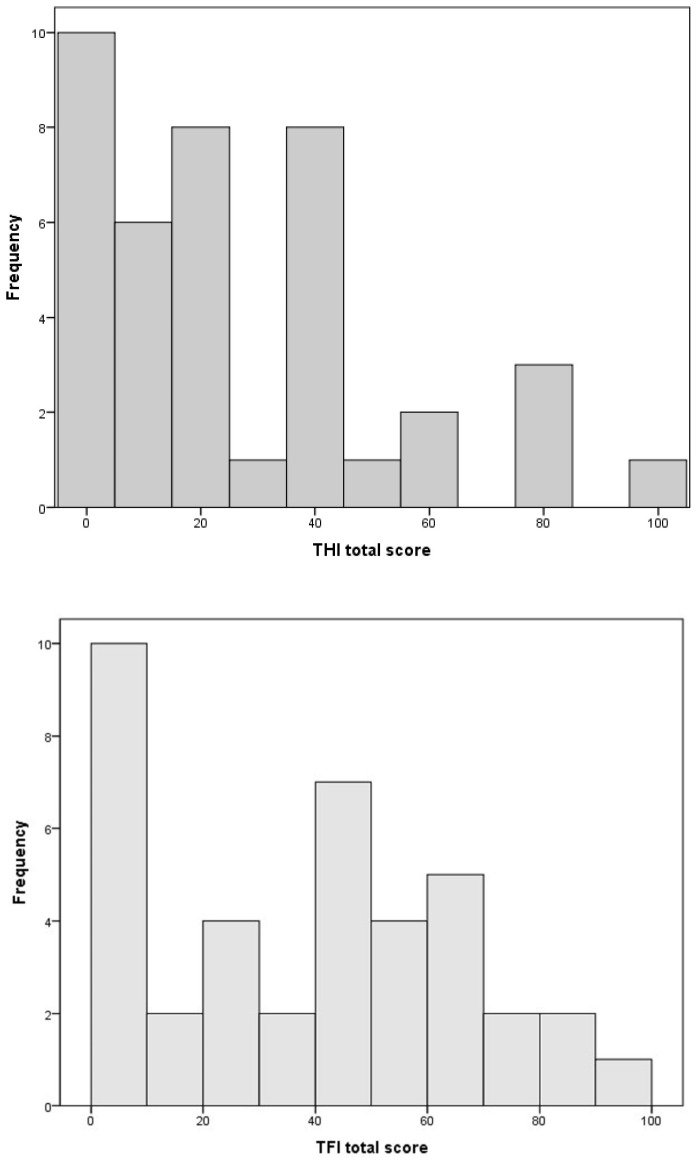
Distribution of the TFI and THI scores at baseline TFI (tinnitus functional index). THI: tinnitus handicap inventory.

The prevalence of tinnitus complaints pre-implantation was 58.3% (42/72 subjects). All the patients with tinnitus suffered from a subjective, non-pulsatile form. The mean duration of the tinnitus was 10.9 years (±10.2, range 1 to 35 years). The mean severity of the tinnitus was significant according to the TFI questionnaire (total mean score of 38.1 (±27.6, range 0 to 93)), and mild when measured by the THI (total median score of 22 (range 0 to 96)). The scores are displayed in Figure 2. The tinnitus was most frequently described as a noise-like sound (54.8%, *n* = 23), ipsilateral (28.6%, *n* = 12) to the ear considered for implantation, or bilateral (54.8%, *n* = 23), and with a mid-frequency pitch (38.5%, *n* = 15). In total, 12 patients (28.6%) indicated that their tinnitus could be masked by environmental sounds, while 12 out of 34 (35.3%) patients who had a hearing aid before implantation experienced an on/off phenomenon, whereby the tinnitus was suppressed while wearing the hearing aid and immediately returned to baseline after taking off the device. Three of them exhibited some form of RI (in one patient, lasting more than 30 min).

The baseline characteristics of the CI population are shown in Table 1, subdivided according to the presence of tinnitus before implantation. For the groups of patients with and without tinnitus pre-implantation, no significant differences could be identified concerning demographic or hearing-loss characteristics.

### 3.3. Effect of Implantation

Postoperative data at the 3- and 6-month follow-ups were collected from 30 (71.4%) and 27 (64.1%) tinnitus patients, respectively. Not all tinnitus patients completed the questionnaires at all different time points, usually due to practical considerations.

Using a cut-off value of 13 points as a clinically meaningful change in TFI score, 60% (18/30) exhibited a clinically significant tinnitus improvement at 3 months after implantation, compared to pre-implantation, with the subjectively largest improvement recorded close to the time of activation of the implant, approximately 4 weeks after surgery. At the 6-month evaluation, the remitter rate was 70.4% (19/27).

**Figure 3:**
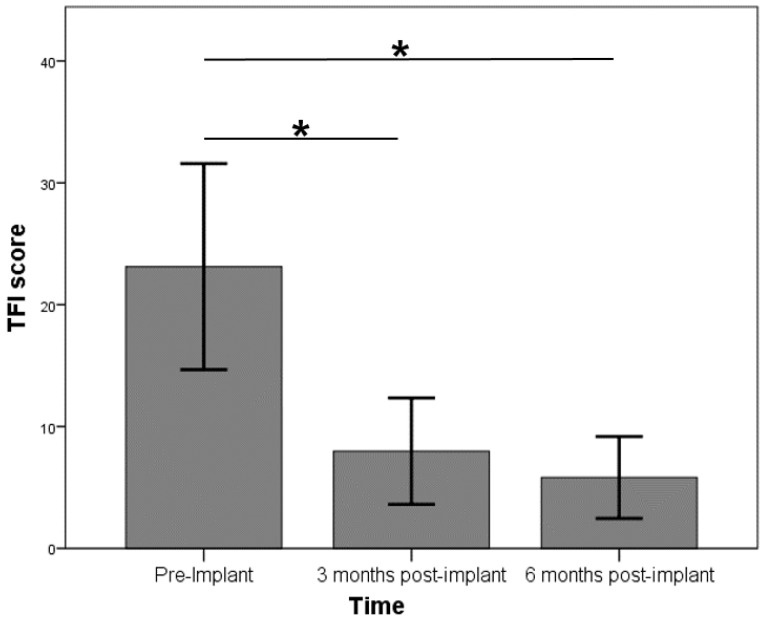
Effect of implantation on tinnitus: average TFI score at baseline and 3 and 6 months after implantation. Statistically significant differences are marked by an asterisk. TFI: tinnitus functional index.

Scores for the change-in-tinnitus questionnaire across the three testing time points in the overall group with tinnitus before implantation were evaluated. Only patients with full data sets were included for analysis. There was a statistically significant difference in TFI score over time using the Friedman test [*χ^2^*(2) = 28.382, *p* < 0.001] (Figure 3). Post hoc pairwise comparisons revealed significant differences between the scores at baseline (pre-implantation) and at 3 months (*Z* = −3.744, *p* < 0.001) and between baseline and 6 months post-implantation (*Z* = −4.349, *p* < 0.001). No significant differences in TFI scores were seen between 3 and 6 months after implantation at follow-up (*Z* = −0.085, *p* = 0.932). Average scores evolved from 41.2 (±29.3) at baseline to 13.0 (±18.4) and 10.3 (±14.6) at 3 and 6 months postoperatively, respectively.

Comparable effects were seen regarding the total THI score: a statistically significant difference over time was found [*χ*^2^(2) = 25.268, *p* < 0.001], with post hoc testing revealing significant changes between the baseline and the 3-month scores (*Z* = −3.655, *p* < 0.001) and between the baseline and the 6-month scores (*Z* = −4.198, *p* < 0.001). The THI score again remained stable at follow-ups between 3 and 6 months post-CI (*Z* = −1.530, *p* = 0.126). The median THI scores for the 3 evaluation time points were 24, 4, and 1, respectively. The improvements in THI sub-scale scores (functional, emotional, and catastrophic) were comparable with changes in the global THI score over time.

**Figure 4:**
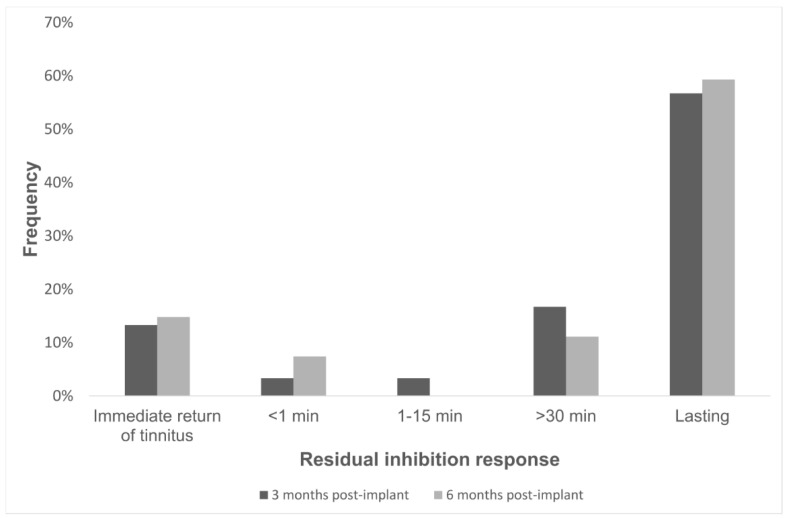
Residual inhibition response after cochlear implantation.

Some form of RI after removal of the CI was present in 24/30 (80%) and 21/27 (77.7%) of patients at 3 and 6 months post-implantation, respectively. This was more than the suppression rate of 3/34 (8.8%) in patients with hearing aids when they removed their devices in the pre-implantation period. The duration of the RI response varied from less than one minute to tinnitus suppression that persisted until the CI was again switched on (e.g., after a night of sleep) (Figure 4).

Based on a description of tinnitus complaints during the evaluation at the 3-month follow-up, newly induced tinnitus appearing during the first postoperative weeks was reported by 5 patients (i.e., 20% of patients with no tinnitus at baseline), and the aggravation of pre-existing tinnitus was reported in 14 patients (i.e., 46.7% of tinnitus patients). Three months after implantation, only one and four subject(s) still experienced tinnitus induction or aggravation, respectively. At the 6-month follow-up evaluation, there remained only 1 patient who exhibited newly induced tinnitus perception, which was of low burden (a TFI score of 13 points).

### 3.4. Associated Factors

The ‘remitter’ and ‘non-remitter’ groups were not significantly different with regard to age, sex, bimodality, or CI electrode configuration, or with regard to their hearing loss characteristics or their tinnitus duration or type (Table 2). There were also no significant differences in hearing preservation, as measured by an LFPTA of unaided post-implantation hearing thresholds or CI performance (aided thresholds and phoneme score at 70 dB SPL).

**Figure 5:**
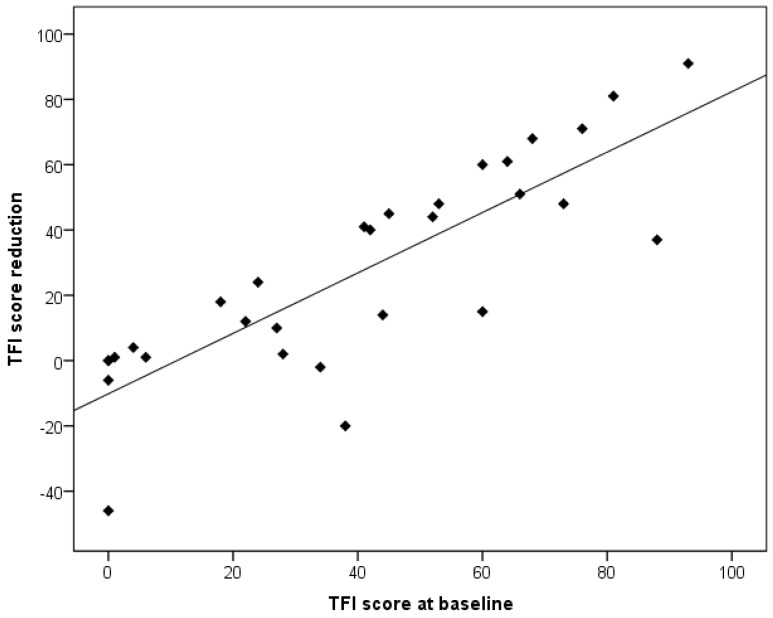
Scatter plot of the correlation between TFI score at baseline and TFI score reduction after 3 months. TFI: tinnitus functional index.

However, there was a significant difference between the remitter and non-remitter groups in terms of baseline TFI scores [*t*(28) = 6.487, *p* < 0.001], as also indicated in Table 2. Furthermore, there was a moderately to strongly significant correlation in TFI and THI scores at baseline, and an improvement in TFI score after implantation (*r* = 0.834, *p* < 0.001 and *r* = 0.653, *p* < 0.001, respectively) (Figure 5). Thus, patients who experienced debilitating tinnitus with a severe impact on daily functioning had a greater chance of experiencing a substantial change in their perception of tinnitus after implantation.

In the subgroup with the (temporary) induction or aggravation of tinnitus post-implantation (*n* = 19), analyses were also performed to identify possible associated factors. This patient group was compared to the group with stable or suppressed tinnitus at the 3-month follow-up or to the group without any tinnitus at all at baseline and at 3 months post-implantation (*n* = 35). We observed a significantly shorter duration of hearing loss [*t*(52) = −2.584, *p* = 0.013] and a higher TFI score at baseline [*t*(52) = 2.512, *p* = 0.015] in the subgroup with worsening tinnitus. Additionally, the aggravation/induction group had more post-lingual than pre-lingual hearing loss [*χ*^2^(1) = 4.349, *p* = 0.037], as well as more ipsilateral tinnitus and hearing complaints [*χ*^2^(1) = 6.100, *p* = 0.047 and *χ*^2^(1) = 10.150, *p* = 0.001, respectively]. The groups did not differ in age, sex, tinnitus duration or type, CI electrode configuration, severity of hearing loss, performance with CI (hearing thresholds or speech understanding), or bimodality or hearing preservation.

## 4. Discussion

The current study assessed the effect of CI on tinnitus perception in a population of 72 subjects receiving implants to treat severe to profound hearing loss.

### 4.1. Effect of Implantation

We observed a statistically significant tinnitus reduction (TFI score difference > 13 points) in 60% of our population at the 3-month follow-up, which remained stable at the 6-month follow-up. Complete suppression rates of 15–83% or a partial reduction of the tinnitus in 46–95% [6,7,9,10,12,16,17,33,34] after CI have been reported in the literature. Our findings are consistent with these results and are quite similar to the effect size of 66.7% that was observed in the prospective study conducted by Kim et al. [11], using a THI score with a clinically meaningful reduction of ≥10. The THI score in our population was reduced by 23.9 points on average at 6 months post-implantation. This result was somewhat larger than the mean range of 13.6–19.5 reported in previous studies [15] and the mean 11.66-point score reduction in a recent meta-analysis [16], although the baseline scores were comparable. As our study is the first to report on TFI score reductions, a comparison with the literature was not possible.

Twenty percent of our patients reported the temporary emergence of new tinnitus complaints immediately after the surgery, whereas 46.7% of the tinnitus patients exhibited a temporary aggravation of symptoms. These numbers were higher than we expected, based on previous results. Usually, induction or aggravation rates vary between 0–23% and 4–26%, respectively [8,15,21,22,23]. These results might have been influenced by recall bias, especially given the possibly increased attentiveness of the study participants regarding possible tinnitus fluctuations as a result of the pre-implantation tinnitus investigations.

However, similar to the observations by Quaranta et al. [6], our finding was that tinnitus aggravation was temporary in the majority of the patients. Tinnitus was still present in only one patient at the last follow-up.

### 4.2. Associated Factors

We included a large variety of possible demographic and hearing-, implant-, and tinnitus-related factors that might be associated with the suppressive effect of CI on tinnitus. We were only able to show a link between the TFI and THI scores at baseline and an improvement in TFI scores after implantation. Our finding that the severity of complaints at baseline is correlated with the implantation effect confirms previous observations [11,17,28], although Ramakers et al. [24] were not able to reproduce this finding.

With regard to preoperative hearing function, our study was not able to identify any significant variables associated with the effect of implantation on tinnitus, which is in accordance with other studies [8,9,10]. In contrast, others have found a lower preoperative speech recognition score [24] and a lower preoperative auditory steady-state response threshold in patients with tinnitus suppression, compared to patients without tinnitus reduction [11]. With regard to postoperative hearing function, no significant factors were found for the tinnitus implantation effect in our study, nor were they found in the study by Kim et al. [11]. However, Ramakers et al. [24] did find a larger deterioration of residual hearing at 250 Hz that was associated with tinnitus softening. A recent retrospective study by Dixon et al. also found worse residual hearing to be associated with higher odds of tinnitus improvement in a logistic regression model of 24 characteristics [17].

In accordance with the literature, no association was found between a tinnitus implantation effect, on the one hand, and age, sex, duration or severity of hearing loss, tinnitus duration [9,10,11,24], surgical approach or insertion depth [11,24], or CI performance (speech discrimination/aided hearing levels), on the other hand [9,10,11], as well as for implant type [35].

As associated factors for the temporary) aggravation or induction of tinnitus after surgery, we found a shorter duration of hearing deprivation, a higher TFI score at baseline, post-lingual onset of hearing loss, and more ipsilateral hearing and tinnitus complaints in this subgroup. This subgroup has not been extensively investigated before, mainly because of its low prevalence. Our finding confirms the results of Pan et al. [8], who reported shorter hearing-loss duration. The latter research also describes an older age at implantation, on average, in patients with induced tinnitus (11/91 patients), compared to those patients whose tinnitus was unchanged or suppressed after CI, or who did not have tinnitus pre- or postoperatively, although this tendency was not statistically significant [8]. Ramakers et al. [24] reported differing, and sometimes contradictory, results. More patients of the female sex, who were of a younger age at onset, and with pre-lingual hearing loss, lower speech recognition scores preoperatively, and lesser deterioration of residual hearing after CI were seen in the group with tinnitus induction after CI (*n* = 7), compared to the group without tinnitus induction after CI (*n* = 43). However, this was again investigated retrospectively and while using only descriptive data. Kompis et al. [10] found lower speech-recognition scores post-CI in the newly induced tinnitus group, as had also been previously observed in 1993 [36]. However, this should be seen as a consequential, rather than a preceding factor.

### 4.3. Mechanisms Underlying the Effect of Cochlear Implants on Tinnitus

Numerous hypotheses have been postulated to explain the suppressive effect of CI on tinnitus complaints [6,7,9,11,37,38].

The most classic theory is focused on peripheral auditory input changes. By enhancing their hearing capacities through CI, patients can focus on masking sounds and distract their attention toward auditory stimuli other than the perception of tinnitus. This masking mechanism suggests a short-term and transient effect, lasting only as long as the stimulation occurs. This hypothesis may be supported by our finding that most of the suppressive effects occur immediately after the activation of the implant, which is also supported in the literature [9,38]. The minority of patients exhibiting no residual inhibition after removal of the implant also underscores this hypothesis.

However, increasing research interest is focusing on the possibility of implantation modulating the tinnitus-related activity in the central neuronal system [34,39].

First, since auditory as well as non-auditory (emotional) pathways are involved in tinnitus perception, the improvement in hearing capacity brought by CI may reduce the stress and anxiety experienced by the patient and, thus, improve their tinnitus coping abilities. Consequently, the reporting of a tinnitus handicap in specific questionnaires (TFI and THI) might be significantly reduced, even though the induction of tinnitus might remain unchanged [6,11,23].

Second, direct stimulation of the cochlear nerve might disrupt its disturbed neuronal synchrony or even central auditory processing in general [40]. Moreover, the fact that a large proportion of our study group (80% after three months) exhibited some form of RI after removing the implant could be an illustration of implantation-induced, long-term neuroplasticity changes, even in the absence of cochlear nerve stimulation. RI responses have been reported frequently in tinnitus research [41] and within the CI population, with prevalence figures ranging from 25% to 92% [5,11,21,24,28,42,43]. Moreover, previous studies have shown that bilateral implantation does not provide an additional reduction in tinnitus compared to unilateral implantation [44,45]. Our study could not reproduce this finding; however, this does strengthen the hypothesis that implantation affects central neuronal activity more than masking or peripheral neuromodulation, wherein we would expect lateralized effects. Also, in most cases, the effects of implantation on tinnitus are bilateral.

This suggests that implantation induces bottom-up processes and dynamic neuroplasticity along central tinnitus-related neuronal networks, as has been shown previously for hearing loss [46]. The exact mechanism and the corresponding neuronal correlates still remain under investigation. In a study by Osaki et al. [47], cerebral PET scans were performed on three tinnitus patients after cochlear implantation. During RI, the anterior and superior temporal gyrus were activated, while the right cerebral hemisphere was activated at baseline (tinnitus being ‘on’ without CI activation). Similar findings were postulated by Giraud et al. [48]. More recently, Song et al. [49] described qEEG results in a small study group with SSD, proposing preoperative cortical oscillation measurement to predict the effect of implantation on tinnitus in SSD. They suggest that different functional non-overlapping brain networks are responsible for an improvement in tinnitus intensity (based on the auditory cortex and posterior cingulate cortex), versus tinnitus-related distress (based on the dorsolateral prefrontal cortex, parahippocampus, and orbitofrontal cortex).

To summarize, arguments can be made for masking as well as for central neuroplasticity changes to account for how implantation might influence tinnitus perception. Most likely, both mechanisms play a role to a greater or lesser extent, depending on the individual tinnitus patient. This is possibly supported by the great heterogeneity seen in the clinical expression of tinnitus. However, the high prevalence of RI in our study reflects long-term changes and strengthens the possibility of implantation-induced modifications to central neuronal tinnitus pathophysiology.

### 4.4. Strengths and Limitations of the Study

The added value of our paper in comparison with the available literature is (1) the prospective design, with fixed follow-up times, and (2) the use of TFI as an evaluation method, stated to be sensitive when assessing subtle effects in terms of tinnitus perception [25,26]. Additionally, we analyzed a large variety of possible factors associated with the effect of CI on tinnitus. Also, by including the CI population as a whole, we were able to compare the tinnitus population with patients without tinnitus, and we were able to investigate the development of new tinnitus complaints.

However, our large patient group also incorporated many heterogeneous characteristics and included uni- and bilaterally implanted patients, patients with uni- and bilateral hearing loss and/or tinnitus complaints, and those with pre- and post-lingual hearing losses, among others. These factors might mask specific effects in certain subgroups. An analysis of homogeneous subgroups would be useful to draw definitive conclusions. However, this was not feasible in our study due to the resulting small study samples.

Also, future research can focus on an exploration of the RI response elicited by electrical versus acoustic stimulation [50], to gain more insight into the main mechanism underlying the implantation effect on tinnitus. Subgroups of interest would be a population with electric-acoustic stimulation or patients with hearing aids who showed an RI response pre-implantation. As the latter subgroup only involved three subjects in our study, who were also not extensively surveyed, the current study could not investigate this topic further.

For a complaint as subjective as tinnitus, one should always interpret results with caution and bear in mind a possible placebo effect. However, the proportion of responders in our study appears to exceed the classically reported placebo effect (30% vs. 60% and 70% responders at 3 and 6 months, respectively). Moreover, we would expect an overreporting of tinnitus complaints in the context of a study where there is more focus on tinnitus and where the subjects are more driven toward it. The reported tinnitus suppression rates could, thus, even be an underestimation of the true effect.

The study of effect-associated variables was rather exploratory, focusing on a prospective investigation, outlining the potential effects, the variables, and the observation of residual inhibition, which, in turn, was suggestive of centrally induced changes. However, this implies that the variables described herein cannot be considered independent predictors for the outcome of implantation on tinnitus until this finding is confirmed by subsequent analysis in future studies.

Another limitation of the current study is that the first postoperative evaluation of tinnitus complaints took place three months after surgery. Based on the patient reports, most of the tinnitus modulation occurred closer in time to surgery and implant activation; these changes were, thus, noted in a descriptive, retrospective manner. Future research would benefit from the introduction of earlier assessment time points (e.g., immediately after surgery/activation and/or one week thereafter).

## 5. Conclusions

The prevalence of tinnitus before implantation was 58.3% in our study population. In 60.0% of patients, a significant tinnitus reduction could be obtained at three months after implantation. This effect was correlated with tinnitus severity at baseline. Eighty percent of these patients with tinnitus reduction exhibited temporary, or even long-lasting, suppression after removal of the implant. This indicates the importance of considering sustainable central pathophysiological mechanisms by which CI might influence tinnitus perception compared to immediate effects achieved by enhancing auditory input or the electrical stimulation of the acoustic nerve.

## Figures and Tables

**Table 1:** Baseline characteristics in the population with cochlear implantation.

	Tinnitus (*n* = 42)	No Tinnitus (*n* = 30)	*p*
**Age at implantation (±SD)**	58 yrs (±14)	51 yrs (±21)	0.140
**Sex (n)**			0.873
**Female**	54.8% (23)	56.7% (17)	
**Male**	45.2% (19)	43.3% (13)	
**HL duration (±SD)**	26 yrs (±17)	27 yrs (±12)	0.714
**HL laterality (n)**			0.071
**Unilateral**	11.9% (5)	0	
**Bilateral**	88.1% (37)	100% (30)	
**HL onset (n)**			0.155
**Prelingual**	21.4% (9)	36.7% (11)	
**Postlingual**	78.6% (33)	63.3% (19)	
**PTA Implant Ear (±SD)**	96 dB HL (±15)	98 dB HL (±13)	0.709
PTA Contralateral Ear (±SD) *	87 dB HL (±16)	93 dB HL (±15)	0.177
**LFPTA Implant Ear (±SD)**	86 dB HL (±23)	82 dB HL (±19)	0.464
LFPTA Contralateral Ear (±SD) *	78 dB HL (±21)	80 dB HL (±20)	0.652
**Tinnitus (*n* = 42)**			
**Tinnitus severity**	TFI score 38.1 (±27.6, range 0–93) THI score 22 (range 0–96)		
**Tinnitus main phenotype**	Noise-like sound (54.8%, *n* = 23), ipsilateral to implanted ear (28.6%, *n* = 12) or bilateral (54.8%, *n* = 23), mid-frequency pitch (38.5%, *n* = 15)		
**Tinnitus duration**	10.9 y (±10.2, range 1–35 y)		

* Calculated only for the group with bilateral hearing loss. The average PTA of the contralateral ear in patients with unilateral hearing loss at baseline was 15 dB HL (±2) and the average LFPTA was 7 dB HL (±8). dB HL, decibel hearing level; HL, hearing loss; LFPTA, low-frequency pure tone average of 0.25, 0.5, and 1 kHz; PTA, pure tone average of 0.5, 1, 2, and 4 kHz.

**Table 2:** Comparison of remitter and non-remitter groups at 3 months after implantation: predictive factors for the effect on tinnitus perception.

	Remitters (Total *n* = 18)	Non-Remitters (Total *n* = 12)	*p*
**Age at implantation (±SD)**	63 yrs (±13)	58 yrs (±12)	0.352
**Sex (n)**			0.765
**Female**	55.6% (10)	50.0% (6)	
**Male**	44.4% (8)	50.0% (6)	
**HL duration (±SD)**	22 yrs (±17)	31 yrs (±19)	0.204
**HL laterality (n)**			0.622
**Unilateral**	22.2% (4)	8.3% (1)	
**Bilateral**	77.8% (14)	91.7% (11)	
**HL onset (n)**			0.184
**Prelingual**	11.1% (2)	33.3% (4)	
**Postlingual**	88.9% (16)	66.7% (8)	
**Tinnitus duration (±SD)**	11 yrs (±9)	9 yrs (±12)	0.738
**Tinnitus type (n)**			0.726
**Tonal**	16.7% (3)	25.0% (3)	
**Noise-like**	55.6% (10)	58.3% (7)	
**Other**	27.8% (5)	16.7% (2)	
**Tinnitus laterality (n)**			0.198
**Ipsilateral**	38.9% (7)	16.7% (2)	
**Bilateral**	55.6% (10)	58.3% (7)	
**Unclear**	5.6% (1)	25.0% (3)	
**Electrode configuration (n)**			0.193
**Modiolus hugging**	66.7% (12)	91.7% (11)	
**Straight**	33.3% (6)	8.3% (1)	
**Bimodality (n)**			0.232
**Yes**	44.4% (8)	66.7% (8)	
**No**	55.6% (10)	33.3% (4)	
**PTA Implant Ear baseline (±SD)**	101 dB HL (±16)	100 dB HL (±15)	0.880
**LFPTA Implant Ear baseline (±SD)**	90 dB HL (±22)	94 dB HL (±15)	0.922
**PTA Implant Ear post-CI unaided (±SD)**	101 dB HL (±13)	82 dB HL (±7)	0.096
**LFPTA Implant Ear post-CI unaided (±SD) ^†^**	88 dB HL (±20)	89 dB HL (±11)	0.052
**PTA Implant Ear post-CI aided (±SD)**	26 dB HL (±4)	24 dB HL (±5)	0.221
**Phoneme score at 70 dB SPL post-CI aided (±SD)**	75 dB SPL (±18)	76 dB SPL (±15)	0.895
**TFI total score baseline (±SD)**	58 (±21)	13 (±15)	<0.001 *
**THI total score baseline (±SD)**	42 (±27)	12 (±16)	<0.001 *

* indicates statistically significant results; ^†^ = only measured in a subset of patients, when there was interest in residual hearing; CI, cochlear implantation; dB HL, decibel hearing level; dB SPL, decibel sound pressure level; HL, hearing loss; LFPTA, low-frequency pure tone average of 0.25, 0.5, and 1 kHz; PTA, pure tone average of 0.5, 1, 2, and 4 kHz.

## Data Availability

The data presented in this study are available on request from the corresponding author. The data are not publicly available due to privacy restrictions of the study subjects.

## Abbreviations

BDI	Beck depression inventory
CI	Cochlear implantation
dB	Decibel
Hz	Hertz
LFPTA	Low-frequency pure tone average
RI	Residual inhibition
SNHL	Sensorineural hearing loss
SPL	Sound pressure level
SSD	Single-sided deafness
TFI	Tinnitus functional index
THI	Tinnitus handicap inventory
TSCHQ	Tinnitus sample case-history questionnaire
